# Review of advanced sensor devices employing nanoarchitectonics concepts

**DOI:** 10.3762/bjnano.10.198

**Published:** 2019-10-16

**Authors:** Katsuhiko Ariga, Tatsuyuki Makita, Masato Ito, Taizo Mori, Shun Watanabe, Jun Takeya

**Affiliations:** 1 WPI-MANA, National Institute for Materials Science, 1-1 Namiki, Tsukuba 305-0044, Japan; 2 Department of Advanced Materials Science, Graduate School of Frontier Sciences, The University of Tokyo, 5-1-5 Kashiwanoha, Kashiwa 277-8561, Japan

**Keywords:** interface, molecular recognition, nanoarchitectonics, sensor, thin film

## Abstract

Many recent advances in sensor technology have been possible due to nanotechnological advancements together with contributions from other research fields. Such interdisciplinary collaborations fit well with the emerging concept of nanoarchitectonics, which is a novel conceptual methodology to engineer functional materials and systems from nanoscale units through the fusion of nanotechnology with other research fields, including organic chemistry, supramolecular chemistry, materials science and biology. In this review article, we discuss recent advancements in sensor devices and sensor materials that take advantage of advanced nanoarchitectonics concepts for improved performance. In the first part, recent progress on sensor systems are roughly classified according to the sensor targets, such as chemical substances, physical conditions, and biological phenomena. In the following sections, advancements in various nanoarchitectonic motifs, including nanoporous structures, ultrathin films, and interfacial effects for improved sensor function are discussed to realize the importance of nanoarchitectonic structures. Many of these examples show that advancements in sensor technology are no longer limited by progress in microfabrication and nanofabrication of device structures – opening a new avenue for highly engineered, high performing sensor systems through the application of nanoarchitectonics concepts.

## Review

### Introduction

Detection systems for various chemical, physical, environmental, and biological targets, so-called sensors, have been continuously explored [1–4]. Although their usefulness was recognized even in the early stages of modern science and technology, the importance of sensors has been recently re-evaluated in the context of current research developments. Today, sensors play an important role in technological advancement for various social demands. There are currently many strategies being pursued for the production of functional materials [5–8], the detection of various risks [9–11], environmental remediation including pollution problems [12–14], energy production [15–17], energy and electricity storage [18–20], device technologies [21–23], and biomedical treatment [24–27], and the targets must be detected with high selectivity, high efficiency, environmental friendliness, and with low cost and low emission. Various fundamental areas of science and technology, such as organic synthesis [28–30], supramolecular organization [31–35], physical fabrication [36–38] and biotechnology [39–41], are expected to solve these problems where some additional factors have to be considered in order to achieve a high degree of control over the structure. This is accomplished by two major processes: (i) selective and sensitive recognition of external inputs (stimuli, substrates, etc.) and (ii) efficient logical conversion to outputs (response, energy, products, etc.). Good sensing systems have many contributions regarding the former part. This is why the importance of sensors has been re-recognized in modern sensor technology.

**Figure 1:**
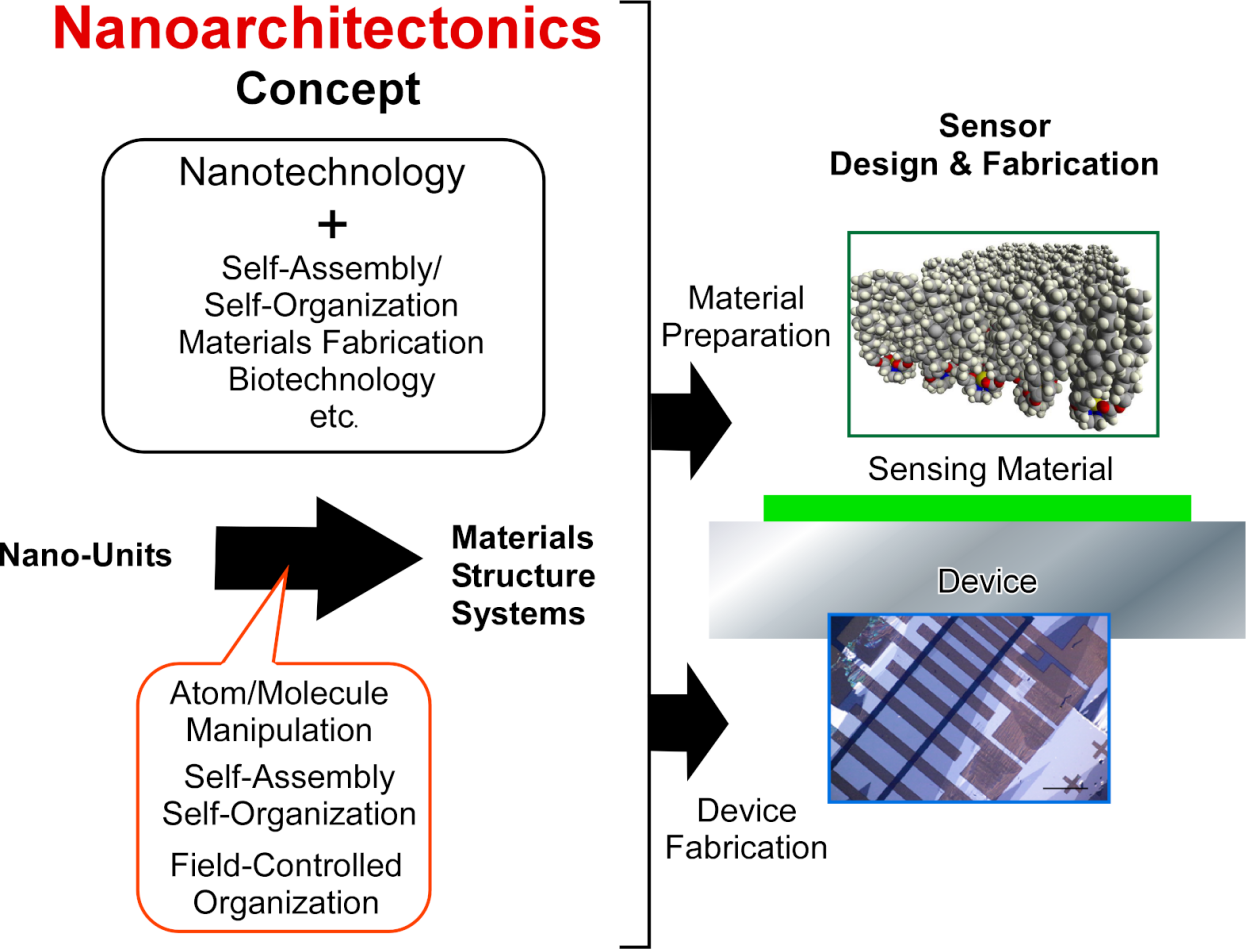
Outline of the nanoarchitectonics concept and its contributions to sensor design and fabrication.

In recent decades, the development of sensor technologies has highly depended on advancements in microfabrication and nanofabrication of device structures. These so-called nanotechnological advancements enable us to prepare sensing devices with various advantageous features with an ultrasmall device size (thus requiring an ultrasmall amount of the target sample), highly integrated connection, and high sensitivity [42–43]. In addition to these nanotechnological advancements in device fabrication, sensing materials for molecular recognition have been continuously explored on the basis of supramolecular chemistry with the aid of synthetic organic chemistry and materials science [44–46]. Therefore, further developments in sensors can be made by the combined efforts in nanotechnology and other research fields including supramolecular chemistry, organic synthesis, and materials sciences. In case of biosensors, contributions from biology play important roles [47–50]. These cross disciplinary collaborations that are necessary for sensor development fit well with the emerging concept of nanoarchitectonics [51–52], which involves a paradigm shift in research efforts to engineer functional materials and systems from nanoscale units through the fusion of nanotechnology with other research fields, including organic chemistry, supramolecular chemistry, materials science and biology. It can thus be said that the future developments of sensors can be supported by the field of nanoarchitectonics [53] (Figure 1).

The nanoarchitectonics concept was originally proposed by Masakazu Aono [54–55]. This conceptual methodology corresponds to the creation of functional materials from nanoscale units through combined processes, including organic synthesis, atomic/molecular manipulation, self-assembly, self-organization, stimuli-based arrangement, and biological treatment, depending on their necessity [56–57]. The high generality of the nanoarchitectonics concept can be applied to a wide range of research concepts, such as materials production [58–60], structure facilitation [61–65], catalysis [66–67], energy technology [68–69], environmental problems [70–71], biological investigation [72–75], and biomedical applications [76–78]. As compared with simple self-assembly processes, nanoarchitectonics is advantageous for architecting hierarchical structures and interfacing between materials and devices. In addition, the fabrication of sensor structures is one of the main outputs of nanoarchitectonics [79–80].

The nanoarchitectonics concept should also include uncertainties related to phenomena that occur on the nanoscale, where thermal and statistical fluctuations as well as quantum effects cannot be avoided [81]. The properties and functions on the nanoscale often result from the harmonization of various interactions. This feature is also found in many biological systems in which functional molecules harmonize under unavoidable thermal fluctuations. The nanoarchitectonics approach and biological processes thus share many of the same features [82]. Therefore, the design and fabrication of biosensors based on the nanoarchitectonics concept may have many particular advantages.

In this review article, we first discuss several examples of recent progress in sensor systems whose advanced nanoarchitectonic design and fabrication allowed for better performance. The examples are roughly classified according to the sensor target, such as chemical substances, physical conditions, and biological phenomena. In the following sections, advancements employing nanoarchitectonic motifs, including nanoporous structures, ultrathin films, and interfacial effects for sensor functions, are discussed. Based on these descriptions, we hope that we can impress the importance upon the advancements of sensor functions with nanoscale control, and especially the importance of nanoarchitectonics in the improvement of these concepts.

### Recent examples of advanced sensors

Advancements in sensor capabilities, including sensitivity, selectivity and usability, can be accomplished by ultrafine design of device mechanisms and sensing material structures. Both the device and sensing material design can be accomplished with a combined concept, nanoarchitectonics, derived from nanotechnology (mainly for the device) and supramolecular chemistry and others (mainly for the sensing materials). For example, Osica recently reported sensor systems for selective acetone vapor detection [83–84]. The prepared systems are supported by two separate innovations, a membrane-type surface stress sensor as a novel nanomechanical device and a highly networked capsular nanoarchitecture of silica–porphyrin hybrid as the sensing material. Not limited to this particular case, innovations from both the device side and the materials side for improved sensors has been continuously pursued.

#### Sensors for chemical substances

**Figure 2:**
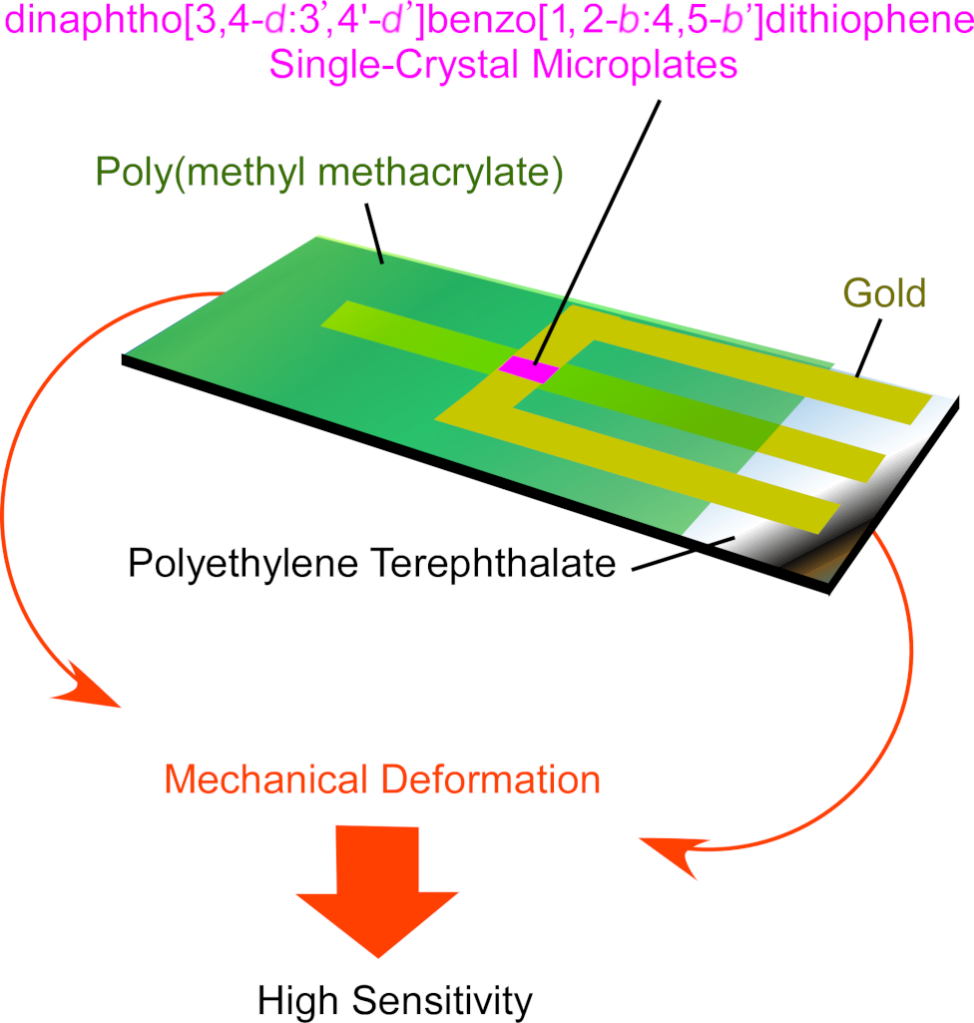
A water-gated bio-organic transistor with odorant binding proteins for the discrimination of chiral substances.

Mainly due to the high demand to solve environmental problems, vapor sensors and gas-phase chemical sensors have been actively researched. Tang and co-workers accomplished drastic improvement of sensitivity of H_2_S gas detection by mechanical deformation of ultrathin single crystals of dinaphtho[3,4-*d*:3’,4’-*d*’]benzo[1,2-*b*:4,5-*b*’]dithiophene in organic field effect transistors [85] (Figure 2). At the tensile state of the crystals, the sensitivity to H_2_S gas at 1 ppm increased by 400% as compared to the original unstressed state. Upon exposure of the sensor crystals to H_2_S gas, the adsorbed H_2_S gas molecules induce a current between the source and drain. Changes in the intermolecular packing of the sensing organic crystals may cause more exposure of active sites to H_2_S gas and dramatic shifts of mobility, resulting in unexpectedly high sensitivity. This example indicates that the delicate modulation of nanoarchitectures can improve chemical sensor capabilities.

As an example of nanoarchitectonics effects between multiple components in sensing materials, Chen, Shi, and co-workers demonstrated highly sensitive resistance-based NO*_x_* gas sensors incorporating a dispersed composite of Co_3_O_4_ nanoparticles in black phosphorous thin films [86]. The composite structures were engineered by functionalization of black phosphorous nanosheets with branched polyethylenimine to which Co_3_O_4_ nanoparticles were included with a hydrothermal process. The sensor composite structures showed ultrahigh sensitivity and a fast response to NO*_x_* gas at room temperature in air, leading to a low detection limit even down to 10 ppb, probably due to the synergic effects of the unique electronic conduction of black phosphor and the heterostructure of the Co_3_O_4_ nanoparticles.

The inclusion of other processes, such as catalytic reactions and fluorescence quenching, often improves sensor capabilities through component nanoarchitectonics. Imanaka and co-workers used a combustion process induced by a precious-metal-free CeO_2_–ZrO_2_–ZnO catalyst for CO gas detection [87]. The semiconducting (p-type) La_2_CuO_4_-loaded CeO_2_–ZrO_2_–ZnO catalyst has a small heat capacity and dramatically increases the temperature of the Pt coil, resulting in a highly sensitive sensor signal. On the other hand, the n-type Sm_2_CuO_4_-loaded CeO_2_–ZrO_2_–ZnO catalyst is advantageous when rapid response and low temperature operation are required. The selection of nanoarchitectonic component materials in sensing units can be used to optimize sensing performance according to usage.

Luminescent xerogel-based sensors for amine vapors were reported by Hanabusa and co-workers [88]. The xerogels used in this sensor system were prepared with fluorescent gelators containing a tris(β-diketonato) complex with appropriate metals. The presence of amines can be found through fluorescence-quenching efficiencies of the thin layer films of the gel materials. The prepared films are most sensitive to the detection of tertiary amines.

**Figure 3:**
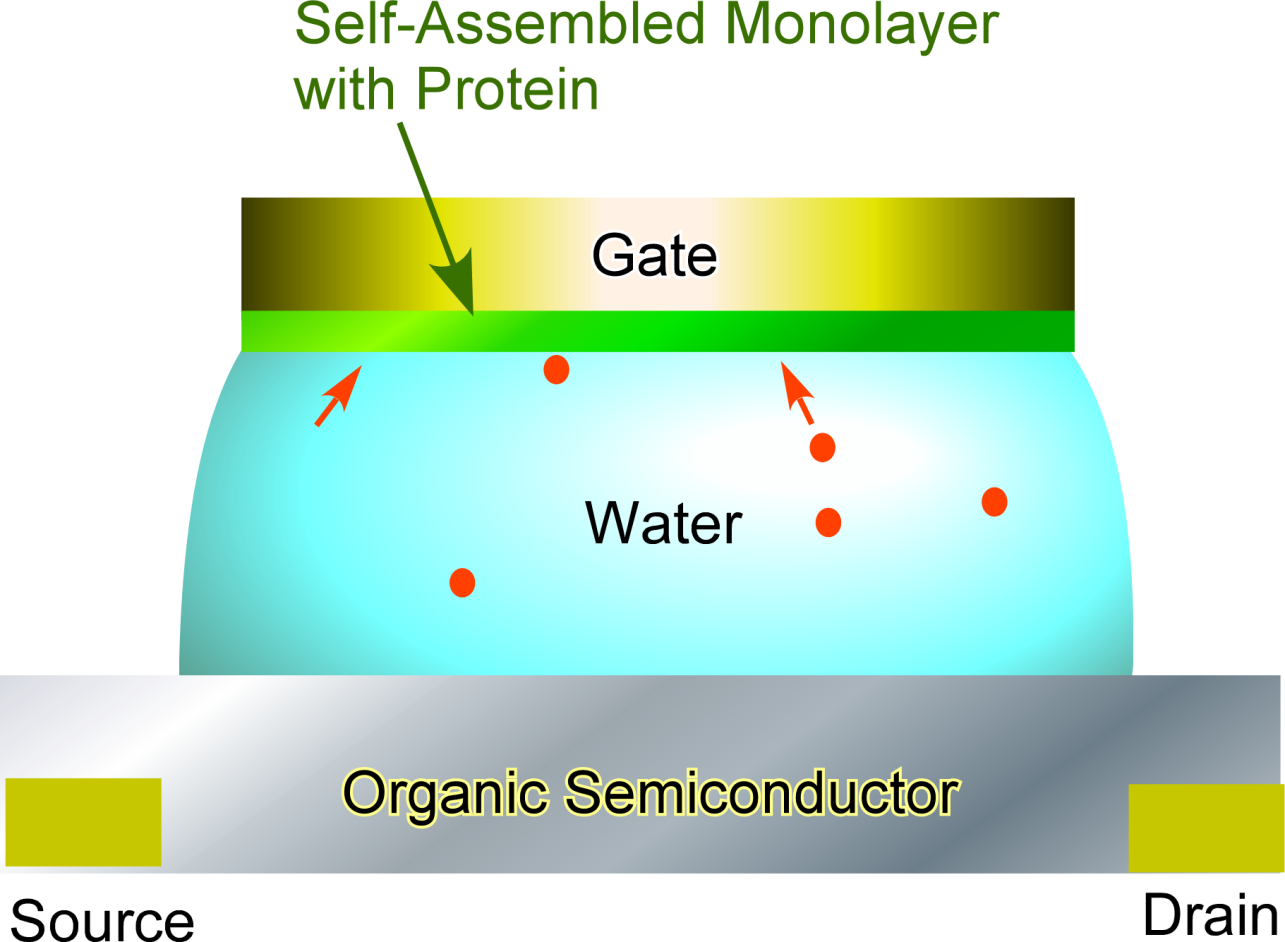
Highly sensitive humidity sensor based on a triboelectric nanogenerator device where nanochannels in the membrane adsorb moisture, which results in the generation of internal stress.

The discrimination and sensing of chiral substances are regarded as a more difficult task because chiral molecules have identical properties except for their optical activity. As recently reported by Kondo et al., the use of chiral receptors is the key to discriminate chiral substances [89]. They used tetraamide-based receptors having chiral ʟ-serine and ʟ-threonine to discriminate enantiomers of *N*-acetyl amino acid anions through ratiometric fluorescence analysis. Torsi and co-workers adopted odorant binding proteins to discriminate chiral substances [90]. They immobilized odorant binding proteins to the gate of a water-gated bio-organic transistor (Figure 3). In this construction, the source and drain patterned substrate was covered with p-type poly[2,5-bis(3-tetradecylthiophen-2-yl)thieno[3,2-b]thiophene], a water droplet and a Au-plate modified with the odorant binding protein as a gate. Enantiomers of odorant carvone could be clearly discriminated by this sensing system. The capacitance changes may be caused by the binding of the odorant to the protein accompanied with the derivation of the free-energy and conformational changes. Such capacitance-modulated transistors would be useful for molecular sensing with weak interaction and faint differences.

Kim and co-workers fabricated sensor arrays that were engineered with fluorescence dyes and cucurbit[*n*]urils (*n* = 6, 7 and 8) as host systems [91]. The obtained sensors were used for sensing biogenic amines with the aid of principal component analysis. This nanoarchitectonics strategy could be applied for the sensing of various bio-related substances and may become useful for diagnostics of diseases such as cancer.

**Figure 4:**
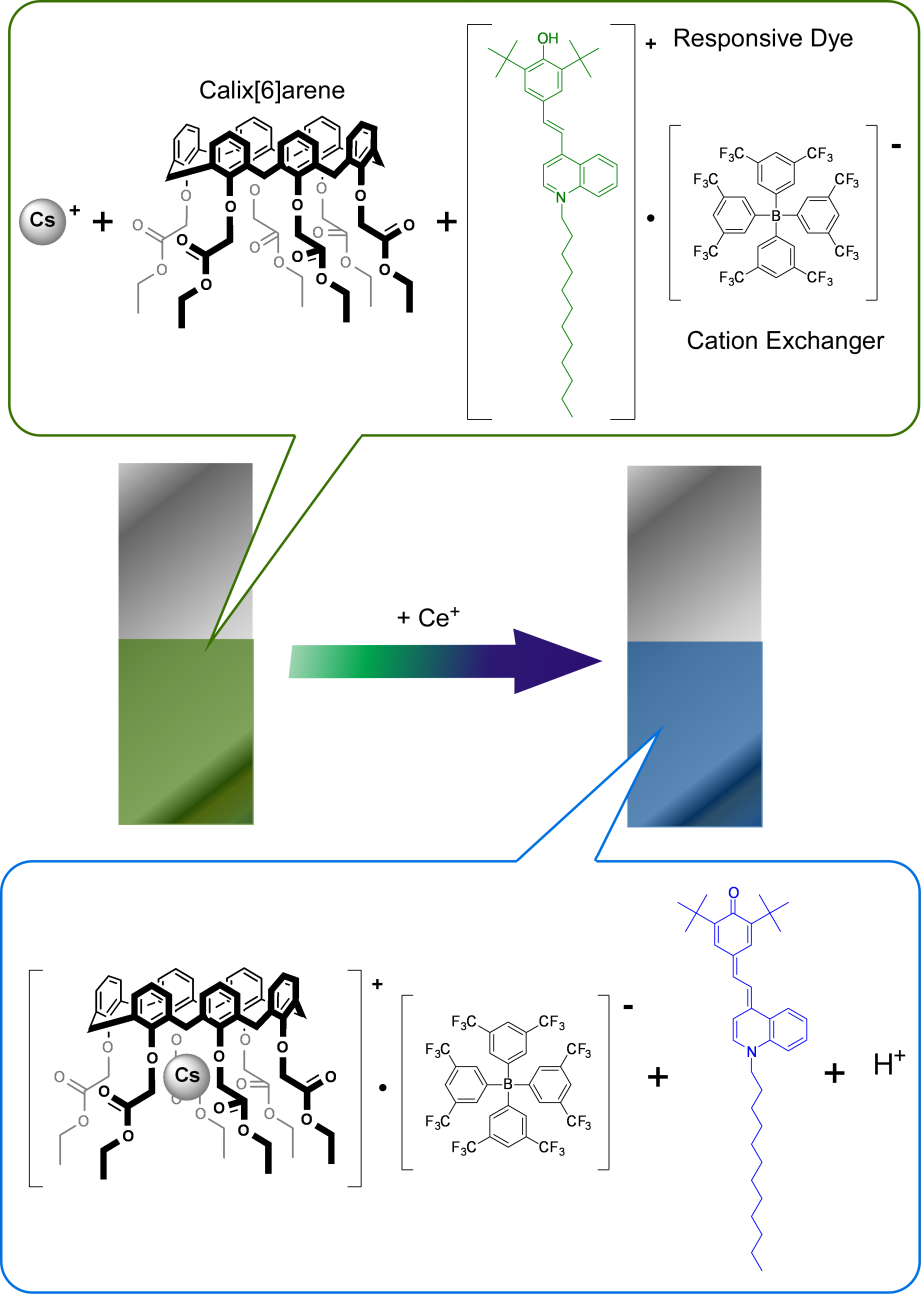
An optode sensor to visually detect cesium ions in domestic water and seawater, comprised of a calix[6]arene derivative, responsive dye KD-M1337, and cation exchanger.

Sensors that are used to detect environmental risks mostly require detection of metal ions and toxic ions. Akamatsu et al. developed an optode-type sensor to visually detect cesium ions in domestic water and seawater [92] (Figure 4). The detection of radioactive cesium species becomes a serious demand after a nuclear plant explosion event, but radioactivity measurements do not always work with high areal resolution. The detection of cesium ions themselves with very high resolution would be useful together with radioactivity analysis. Cesium ion sensing using a film-type optode and nano-optode sensors would satisfy the former requirements. The optode sensors designed using nanoarchitectonic concepts incorporated a calix[6]arene derivative, responsive dye KD-M1337, and a cation exchanger sodium tetrakis[3,5-bis(trifluoromethyl)phenyl]borate. The binding of cesium ions to the calix[6]arene derivative shifts the equilibrium, resulting in color changes even in domestic water and seawater. Sonicating this optode mixture provides nano-optode sensor particles at a diameter of approximately 100 nm, which is a material capable of detection of cesium ions in sub-micromolar levels.

Use of 2D and layered materials in nanoarchitectonics for ion sensors is also investigated. Ruiz-Hitzky et al. reported the fabrication of potentiometric sensors for alkali-ion detection using clay materials intercalated with silacrown ethers, dimethylsila-14-crown-5 and dimethylsila-17-crown-6 [93]. The nanoengineered montmorillonite-based intercalation materials were included in poly(vinyl chloride)-based electrodes for potentiometric sensors towards alkali-metal ions in solution. Ultrasensitive sensors for mercury ions were prepared by Li et al. who engineered suspended atomically thin black phosphorus between the source and drain electrodes [94]. Due to the avoidance of substrate scattering, the sensors with bridged black phosphorus exhibit a much improved signal-to-noise ratio in mercury ion detection with a detection limit of 0.01 ppb and a very short detection time constant of 3 s. This nanoarchitectonic design can maximize the intrinsic potential of black phosphorus and other materials.

#### Sensors for physical conditions

Not limited to particular chemicals, the sensing of general external environments such as pH, humidity, pressure, and magnetic field is undoubtedly important. Spanu et al. reported sensitive pH sensors based on organic charge-modulated field-effect transistor structures with 6,13-bis(triisopropylsilylethynyl)pentacene [95]. The fabricated sensors have a super-Nernstian sensitivity and reference-less nature. This organic charge-modulated field-effect transistor mechanism is attributed to the variation of the threshold voltage in the organic field-effect transistor induced by charge variation upon the presence of a charge (protonation, etc.) on the sensing area. The sensitivity of the nanoengineered sensors is easily tunable by adjusting geometry-related parameters.

For practical uses, sensors are not always used in ideal conditions. Especially, their use in dynamic human life, including health monitoring and medical applications, require consideration of bending and deformation according to typical human motions. Someya and co-workers developed transparent bending-insensitive pressure sensors [96]. They nanoengineered pressure sensor materials from composites of carbon nanotubes and graphene with a fluorinated copolymer, vinylidenefluoride-tetrafluoroethylene-hexafluoropropylene, and an ionic liquid, 1-butyl-3-methylimidazolium bis(trifluoromethanesulphonyl)imide, through an electrospinning process. The prepared sensor can only sense normal pressure without significant disturbance up to a bending radius of 80 µm. This sensor system could be applicable for demands requiring the evaluation of small normal pressures even on dynamic surfaces such as natural tissues and is expected to be useful for in situ biomedical digital monitoring, such as palpation for breast cancer.

**Figure 5:**
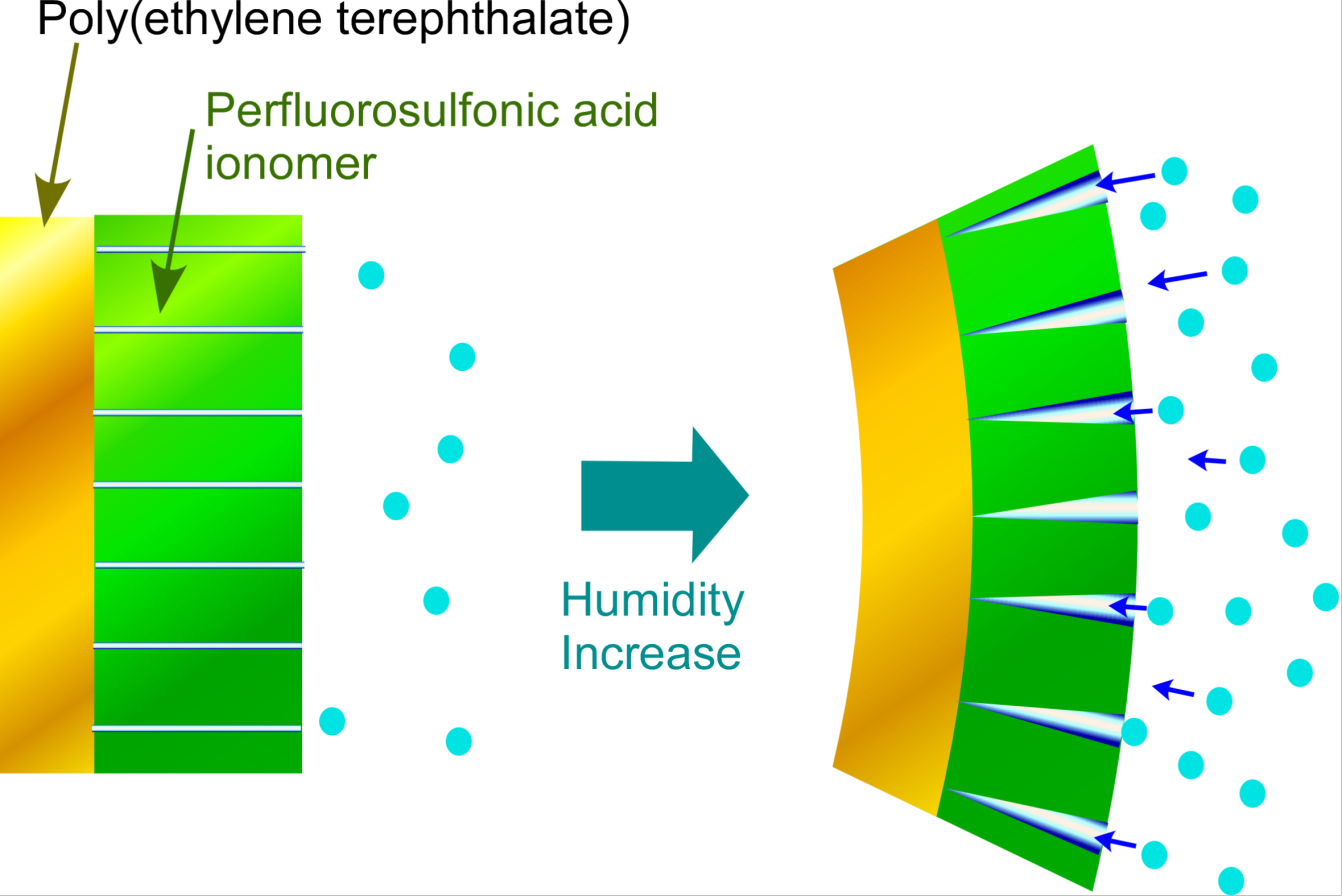
An electrolyte-gated organic field-effect transistor with anti-bisphenol A antibody. The addition of bisphenol A induces the removal of the antibody from the sensor surface, resulting in an increase in the drain current.

Triboelectric nanogenerators to convert mechanical energy to electricity have recently been given much attention as self-powered systems. These systems can be designed using nanoarchitectonic principles with various sensing materials to form energy harvesting self-powered sensors [97–98]. Chen and co-workers introduced a perfluorosulfonic acid ionomer as a water-vapor-driven actuation material for a triboelectric nanogenerator device to realize a highly sensitive humidity sensor [99] (Figure 5). The reaction of the sensing materials to humidity results in electrical changes for sensing. The perfluorosulfonic acid ionomer membrane has perpendicularly extended nanochannels that can adsorb moisture. Under relatively high humidity conditions, the adsorption of water molecules expands the nanochannels resulting in internal stress generation. These changes can be sensitively detected by the triboelectric nanogenerator. At the same time, the collection of such electrical signals can work as energy harvesting devices from wind and raindrops. Similarly, Liao, Wang, and co-workers used a triboelectric nanogenerator system of thin films of fluorinated ethylene propylene to fabricate self-powered wind sensors operating in free-standing mode (anemometer triboelectric nanogenerator) and single-electrode mode (wind vane triboelectric nanogenerator) [100]. The former mode can be used for analysis of wind speed with less energy consumption and the latter one provides an accurate measurement for the wind direction. The wireless monitoring of these responses could contribute to large-scale climate monitoring.

Although various living creatures, including bacteria, insects, birds, and sharks, can sense magnetic fields for orientation and navigation, humans are basically insensitive to magnetic fields. The human detection of magnetic fields can be realized using electro-skin-type sensors for magnetic fields. Makarov and co-workers developed giant magnetoresistive sensors in foil form having high flexibility and mechanical durability [101]. For giant magnetoresistive materials, multilayer structures of Co/Cu and permalloy/Cu multilayers (permalloy = Ni_81_Fe_19_) were engineered on ultrathin polyethylene terephthalate foils. The prepared sensors are extremely flexible (bending radii <3 µm) and light weight (≈3 g m^−2^). They are wearable and act as a magneto-sensitive skin with navigation and touchless control capabilities.

#### Biosensors

**Figure 6:**
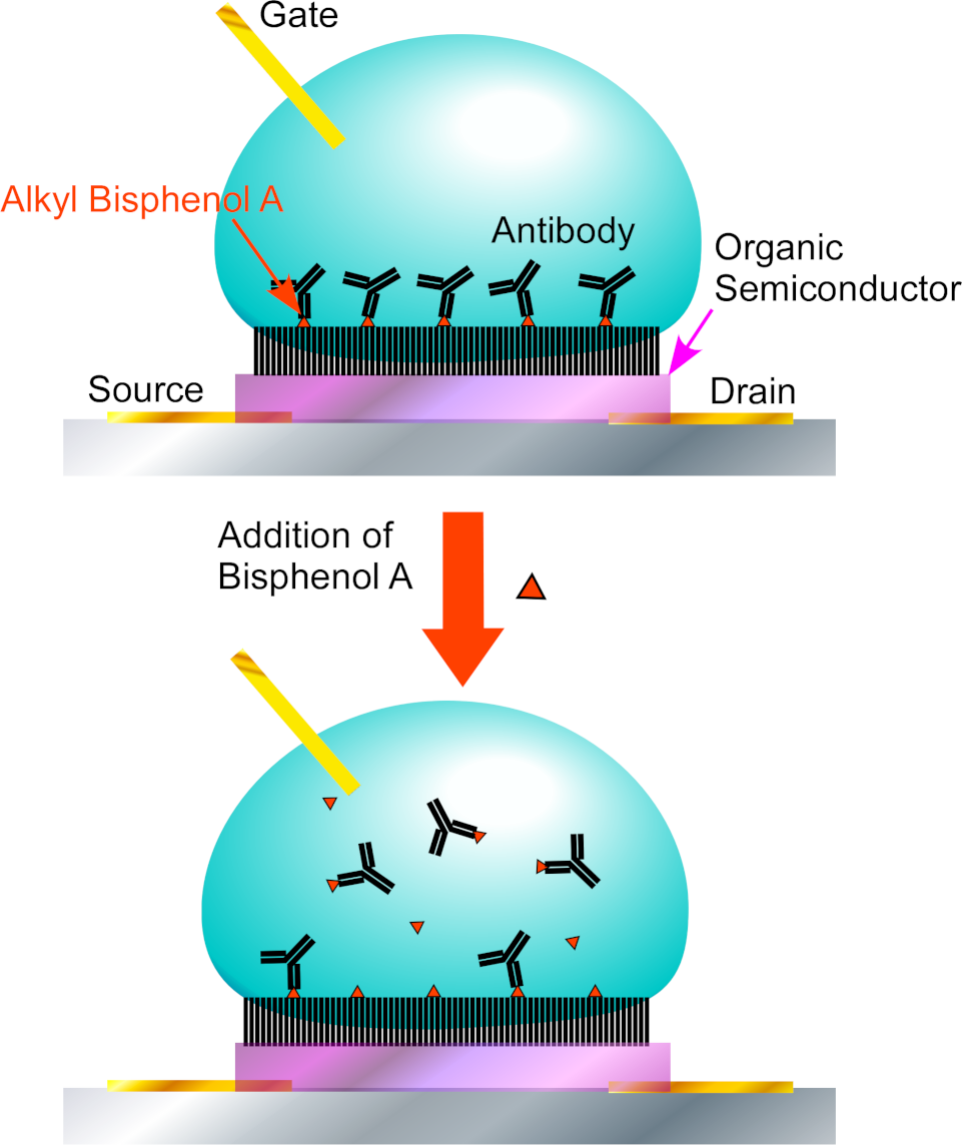
A field-effect transistor with a monolayer of pentathiophene-type organic semiconductor for melamine detection where the monolayer structure of the semiconductive layer provides better sensor performance with long-term stability and high sensitivity.

Because biosensors can provide crucial contributions to human life, medical, and health monitoring, the development of biosensors has received significant attention. For example, for the detection of bisphenol A, which is suspected as an endocrine disruptor, Piro et al. produced a nanoarchitectonic electrolyte-gated organic field-effect transistor with poly(2,5-bis(3-tetradecylthiophen-2-yl)thieno[3,2-b]thiophene) as an organic semiconductor co-crystallized with an alkyl derivative of bisphenol A as a hapten [102] (Figure 6). Upon binding of the anti-bisphenol A antibody, the output current of the transistor first decreased. The addition of bisphenol A induced the removal of the antibody from the sensor surface through competitive binding, resulting in a capacitance increase accompanied with an increase of the drain current. The switching-on signal response of this system is in the nM range of concentration threshold for bisphenol A detection. This sensitivity is sufficient for the detection and monitoring of this persistent pollutant in drinking water.

As mentioned previously, the discrimination of chiral substances is a rather tough goal in sensor design. However, the detection of chiral amino acids is an unavoidable matter in technologies related to protein metabolism, food products and pharmaceuticals. Such difficult goals can be achieved through a nanoarchitectonics approach, namely molecular imprinting [103–105]. Qiu and co-workers developed sensors for chirality detection of amino acid guests using an organic electrochemical transistor with a poly(3,4-ethylenedioxythiophene)/poly(styrenesulfonate) (PEDOT/PSS) system modified with molecularly imprinted polymer films [106]. The selectivity factor of ʟ-tryptophan over ᴅ-tryptophan and that of ʟ-tyrosine over ᴅ-tyrosine were 11.6 and 14.5, respectively.

Life activities are also important targets in biosensor technology. Someya and co-workers developed a highly flexible organic amplifier to detect weak biosignals [107]. A highly conductive biocompatible gel composite made from multiwalled carbon nanotubes and aqueous hydrogel was integrated into a two-dimensional organic amplifier. The biocompatible nature of these nanoarchitectures is advantageous to favorably interface the bio-tissues and device electrodes. The dynamic motion of a living heart can be sensitively monitored without mechanical interference. This enables the direct evaluation of epicardial electrocardiogram signals with an amplification factor of 200. This idea can be expanded to various practical sensing demands such as temporal monitoring during medical surgery and long-term implantable monitoring. Ingebrandt and co-workers also reported biosensors to monitor electrophysiological activity of the cardiac cell line HL-1 [108]. The sensing system is based on organic electrochemical transistors with PEDOT/PSS materials produced with a wafer-scale process, which is known to be useful for transducing and amplifying biological ionic signals.

In certain cases, device architectures and cell nanoarchitectures have to be well-matched for better sensor performance. Hsing and co-workers investigated the difference in sensing signals between tightly packed colorectal adenocarcinoma cells and leaky nasopharyngeal carcinoma cells using biosensors based on an organic electrochemical transistor [109]. The biosensor performance depends on the impedance of whole the system, including the transistor devices and monitored cells. Since cell packing affects the sensor signal, the optimum design of such cell sensors should be tuned according to cell packing. In order to evaluate the paracellular characteristics of tightly packed cells, a large-sized organic electrochemical transistor is advantageous. On the other hand, the high frequency related information of leaky cells can be effectively monitored by smaller organic electrochemical transistors.

Biosensors based on the electrical double layer gated AlGaN/GaN high electron mobility transistors were used to dynamically monitor changes in the transmembrane potential, as reported by Lee, Wang, and co-workers [110]. Here, circulating tumor cells of colorectal cancer together with cellular bioelectric signals were investigated. The proposed sensor design would also be useful for the rapid screening of diseases as a point-of-care diagnostic tool. Owens and co-workers developed organic field-effect transistor systems with PEDOT/PSS materials for the detection of lactate [111]. Enhanced lactate production was detected for cancer cells because of their promoted activity of glycolytic metabolism. These nanoengineered miniaturized biosensors would be useful for the continuous monitoring of tumor status in cancer patients.

### Advancements in nanoarchitectonic motifs

In the previous sections, several examples of advanced sensor systems were reviewed according to sensing targets, chemical substances, physical conditions, and biological activities. The importance of a high degree of structural control (microscopic and nanoscopic levels) both for the sensing materials and the device structures can be found in most of the cases. Advanced sensors are certainly improved by application of nanoarchitectonics strategies. In the following sections, sensor designs are discussed on the basis of nanoarchitectonic structural motifs, such as nanoporous structures and extremely thin nanofilms as well as the highly enhanced molecular sensing capability at interfacial structures.

#### Porous structures

One of the most highly effective methods to improve the sensitivity of sensors is the enhancement of the surface (interfacial) area for facile contact between the sensing target molecules and sensor device material. High surface area materials such as integrated structures and nanoporous materials can be obtained by molecular self-assembly [112–114] and template synthesis [115–119]. For example, various sensors with self-assembled fullerene materials and their carbonized materials as sensing structures were reported for aromatic gas vapors [120–121] and carbonized particles [122]. Mesoporous carbons were used for sensing of tannins in acidic aqueous environment with highly cooperative adsorption in the mesochannels [123]. The nanoarchitectonic construction of carbon nanocages with high surface mesoporous structures [124] was integrated with electrospun polymer fibers and resulted in a highly sensitive sensing material for aniline vapor [125]. Layer-by-layer structures of mesoporous carbon capsules can work as sensing membranes capable of selectively sensing through the doping of secondary sensing units [126].

As an emerging nanoporous material, metal–organic frameworks and porous coordination polymers have received much attention because of the various functional nanoporous structures that can be engineered through self-assembly from selected components [127–130]. Pan, Su, and co-workers fabricated metal–organic framework materials with microporous structure and switchable luminescence capability for sensitive water detection [131]. The metal–organic framework sensor was prepared from Zn and (5-(2-(5-fluoro-2-hydroxyphenyl)-4,5-bis(4-fluorophenyl)-1*H*-imidazol-1-yl)isophthalic acid) ligands, the latter of which shows a characteristic excited state intramolecular proton transfer. The adsorption of water molecules into the micropores induces interconversion between the hydrated and dehydrated phase, accompanied by the switching on and off of the excited state intramolecular proton transfer, resulting in sensitive switching between two-color photoluminescence. The sensing films consisting of paper and ZnO could realize a very sensitive water detection with a relative humidity of less than 1% and the detection of trace-level water of less than 0.05%. In addition, the interference from any small molecules other than water is also avoided. The precise nanoarchitectonic control of the structure results in high sensitivity and selectivity.

The nanoporous architectures of metal–organic frameworks can also serve as filters for molecular selection. Fan and co-workers prepared an electrical gas sensor for formaldehyde with high selectivity using the molecular sieving function of zeolitic imidazolate framework structures on ZnO nanorods [132]. Core–shell structures of zeolitic imidazolate frameworks and ZnO nanorods were prepared by direct growth of the framework on the ZnO nanorods. Limitation effects by the framework aperture provided improved selectivity for formaldehyde over the other volatile organic compounds. This nanoarchitectonic strategy using molecular sieving effects of nanoporous frameworks can be applied to other targets of selected molecular size.

#### Ultrathin films

The immobilization of functional materials with ultrathin films such as self-assembled monolayers [133–134], Langmuir–Blodgett films [135–137], and layer-by-layer assembly [138–141] on sensors and related devices is a key nanoarchitectonics step for sensor fabrication. For example, Furusawa et al. immobilized nickel-nitrilotriacetic acid within a self-assembled monolayer on an organic field-effect transistor, which was used for the sensitive detection of small organic acid molecules, such as citric acid [142]. Hattori and co-workers fabricated an ATP/ADP sensitive image sensor by immobilization of apyrase as a self-assembled monolayer on a 128 × 128 pixel array semiconductor CCD-type pH imaging sensor [143]. Although the sensitivity of the prepared sensor is inferior to that of other fluorescence sensors, this sensor nanoarchitectonics approach does not require any labelling procedures. Therefore, it may be useful for the estimation of ATP discharge in damaged cells.

**Figure 7:**
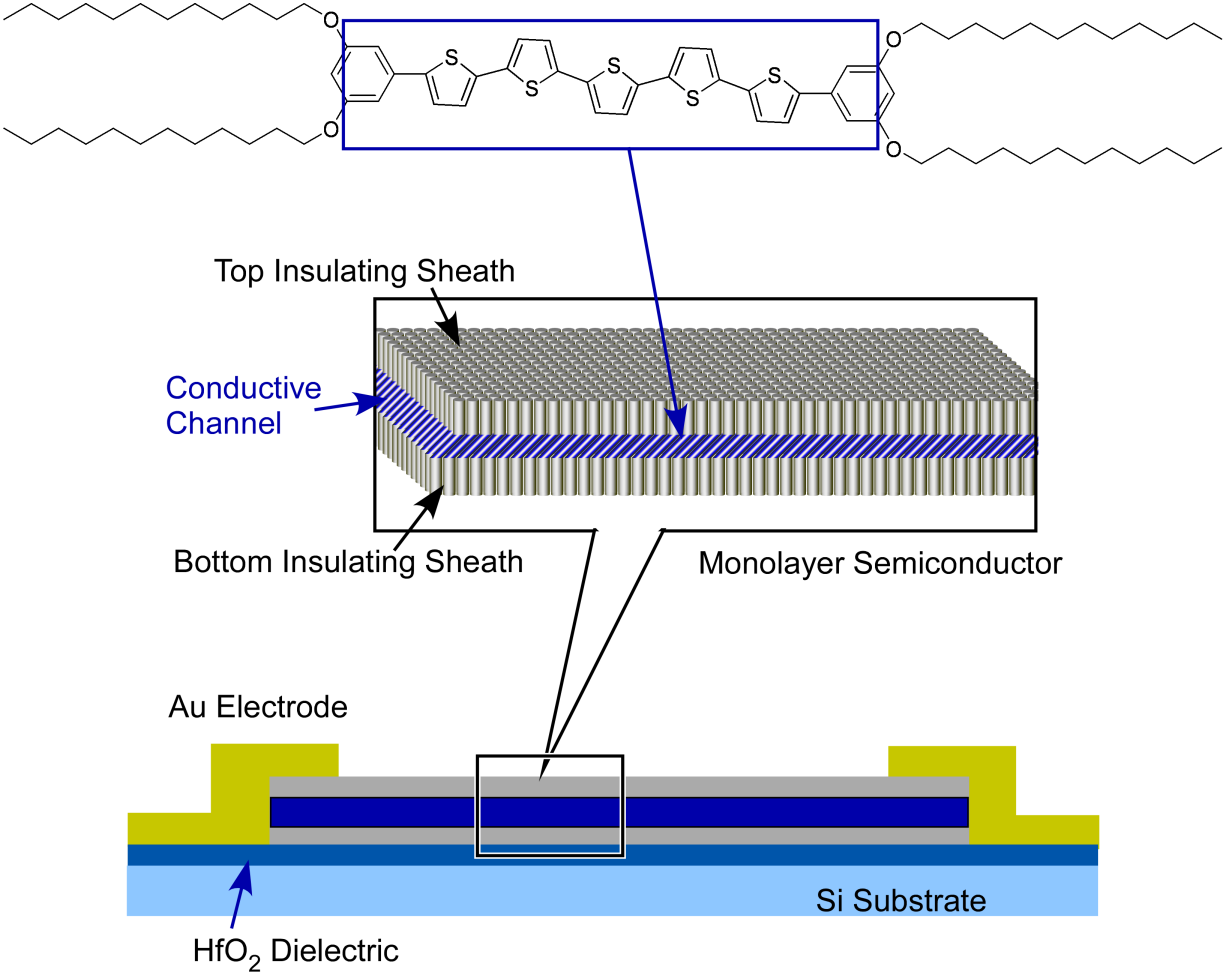
Features of molecular recognition at interfacial media: (i) contacts of phases with different dielectric natures to result in huge enhancement of molecular recognition; (ii) connection of extremely different size events, enabling the manipulation of molecular receptors by macroscopic motion. Adapted with permission from [51] copyright 2015, The Royal Society of Chemistry.

Ultrathin film nanoarchitectures are crucial not only for the facile contact between analytes and the sensor device but also with respect to the carrier mobility for semiconductor-based sensor devices. The enhancement of sensor performance on ultrathin films has been recognized in several recent research efforts. Guo and co-workers fabricated a field-effect transistor with monolayers and multilayers of pentathiophene-type organic semiconductor for melamine detection [144] (Figure 7). The used dialkoxyphenyl pentathiophene derivative has a semiconductive pentathiophene core sandwiched by two insulating C_12_ alkyl chains. Long-range ordered π-conjugated columns in densely packed arrays of the pentathiophene core confine charge carrier transport to one direction. The charge generation and transport can be effectively maximized by this carrier transport confinement. The fabricated field-effect transistor structure was integrated into a microfluidic device. The monolayer structure of the semiconductive layer provided better sensor performance with long-term stability and high sensitivity. The minimum detection limit for melamine was approximately 10 ppb.

The high performance of nanoarchitectonic semiconductive monolayers was also demonstrated by Chan and co-workers who successfully prepared semiconductor monolayer crystals of 2,9-didecyldinaphtho[2,3-b:2’,3’-f]thieno[3,2-b]thiophene on the millimeter scale [145]. The semiconductor crystals encapsulated within poly(methyl methacrylate) exhibited a significantly high mobility (10.4 cm^2^ V^−1^ s^−1^). In multilayer structures, the first layer on interface plays the main role in carrier transport and the layers above simply act as carrier suppliers. The crystal monolayer shows low anisotropy and thermally activated carrier transport. Such characteristics are different from the band-like carrier transport modes in thicker crystals. The fabricated sensor with ultrathin organic semiconductor crystals was an efficient NH_3_ sensor with a detection limit on the 10 ppb level.

#### Specific effect of molecular sensing at interfaces

**Figure 8:**
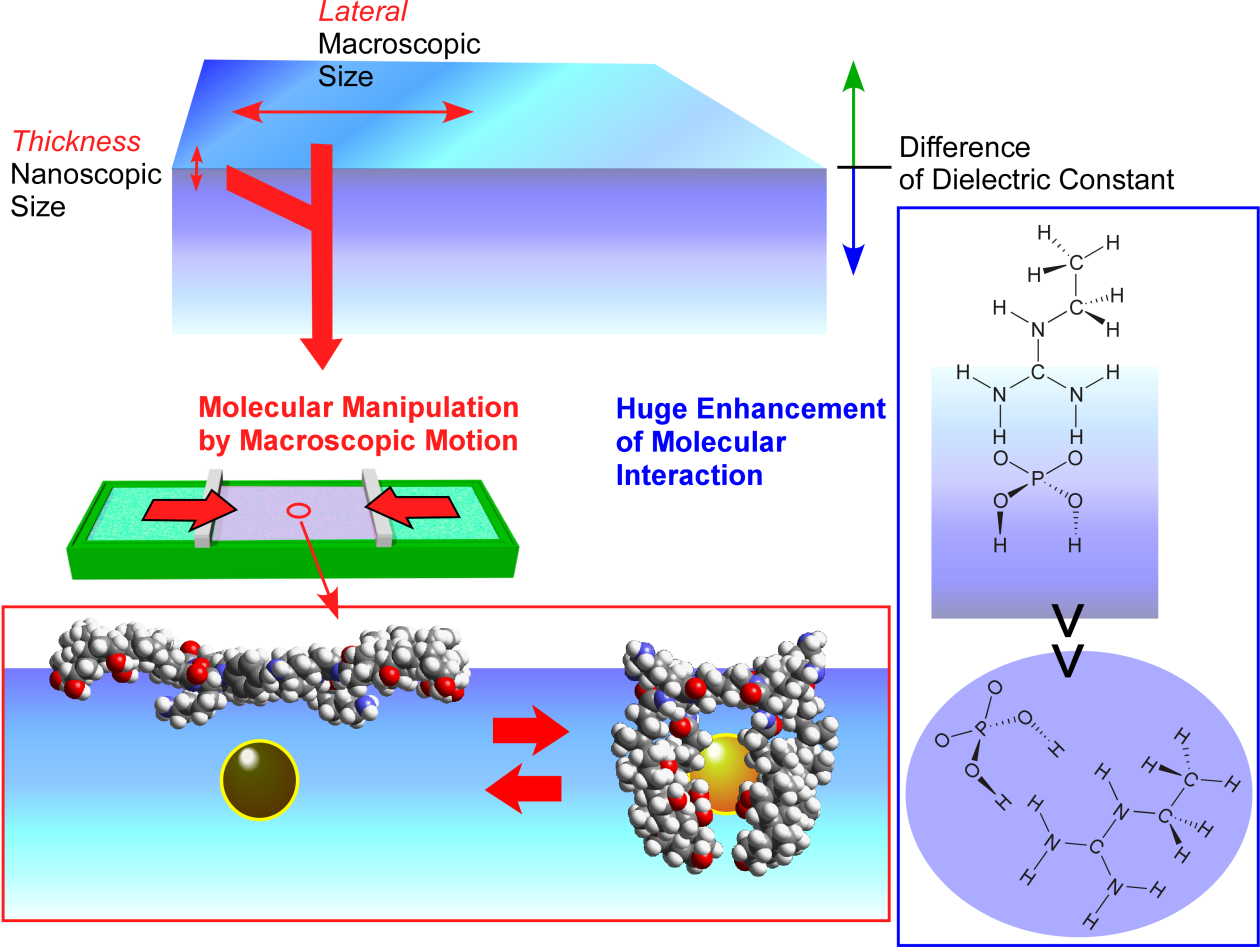
Mode of molecular recognition and sensing: (A) one most stable state between guest and host; (B) switching molecular binding by external stimuli; (c) tuning of receptor structures through continuously shifting molecular conformations for required demands. Adapted with permission from [52] copyright 2016, Springer Nature.

The high surface area nature of nanoporous materials and the ultrathin aspect of monolayer crystals are advantageous for improved sensor performance. These structural features can also be regarded as interfacial nanoarchitectonics. In this section, the scientific basis for molecular sensing (recognition and discrimination) specific to interfacial environments is briefly described and hints for future sensor designs are discussed. Interfacial environments provide two distinct features, (i) contacts of phases with different dielectric natures and (ii) connection of extremely different size events (both along the lateral direction and thickness direction, Figure 8).

Interfaces provide the potential of contact between two different media. Molecules with recognition capability (receptor molecules) prepared through organic synthesis are not always soluble in the aqueous phase and are not appropriate for sensing of water-soluble targets in solution phases. Placing such water-insoluble receptor molecules at a water-contacting interface is crucial to sense water-soluble substances such as important biomolecules. Not limited to this technical requirement, interfacial media have the benefit to greatly enhance molecular recognition capability [146–147].

Systematic research on the comparison of recognition efficiencies of a fixed molecular pair (guanidinium and phosphate) revealed that binding constants change significantly depending on interfacial types [148]. The binding constant between guanidinium and phosphate dispersed in aqueous media is only 1.4 M^−1^ [149]. This kind of molecular recognition based on hydrogen bonding and/or electrostatic interaction is weakened in polar media such as water phase. Chemical species with uneven charge distribution within a molecule are stabilized by solvation with polar solvent molecules, which is highly disadvantageous in the formation of host–guest complexes. However, the incorporation of guanidinium functionality into molecular assemblies such as aqueous micelles and lipid bilayers to place recognition sites at a mesoscopic interface increased the binding constants between guanidinium and phosphate to 10^2^–10^4^ M^−1^ [148]. Furthermore, placing a guanidinium functionality at a macroscopic interface, such as air–water, results in a huge enhancement of the binding constant with aqueous phosphate to 10^6^–10^7^ M^−1^ [150–151]. These facts imply that the molecular sensing capability could be improved by selecting interfacial types and nanoarchitectonics of interfacial structures.

The above-mentioned specific features at the interfacial media were also proved by theoretical calculations based on quantum chemistry [152–154]. Even without direct contact, the low dielectric nature in nonpolar media located close to recognition sites provided positive effects. It is a plausible mechanism regarding how biological molecular recognition occurs in aqueous media [155]. The molecular recognition of small molecules can be accomplished at certain kind of interfacial environments such as cell membrane, inside surfaces of receptors and enzymes, and macromolecular interfaces at DNA and proteins. This mechanism for the enhancement of the molecular recognition capability at interfaces is surely applicable to other molecular recognition pairs and should also lead to highly efficient molecular recognition of various aqueous biomolecules including amino acids [156], peptides [157–159], sugars [160–161], nucleic acid bases [162–163], and nucleotides [164–166] at well-designed interfacial environments. In order to design and fabricate sensors with better performance, interfacial nanoarchitectonics should be crucial factor.

Another feature specific to interfacial environments is the co-existence of extremely different sized structures. At dynamic interfaces, their lateral direction has macroscopic motional freedom but the structural changes in the thickness direction are confined to the nanometer scale. Therefore, macroscopic motions such as compression and expansion can be coupled with nanoscopic conformational changes of molecules embedded at dynamic interfaces [167–169]. For example, dihedral angles of binaphthyl units can be continuously tuned at the molecular level by dynamic compression and expansion of monolayers of tens of centimeters [170]. The digital switching of helicity of binaphthyl units is also possible through macroscopic motion [171]. Furthermore, the control of nanoscopic motions of molecular machines such as molecular catchers [172–173] and molecular motors [174–175] can be accomplished by macroscopic motions at the air–water interface.

The regulation of molecular conformation at interfacial media can be utilized for the tuning of molecular sensing. The structural tuning of an octacoordinate Na^+^ complex of a cholesterol-substituted cyclen with twisting helicity at the air–water interface was used to realize switching recognition selectivity between ʟ- and ᴅ-amino acids [176–177]. Chiral sensing can be tuned by mechanical deformation of the receptor membrane at the interfacial environment. Two-dimensional deformation of cholesterol-substituted triazacyclononane monolayer was used to optimize the discrimination between uracil and thymine derivatives [178–179] that cannot be discriminated by naturally occurring DNA and RNA. Although the structural difference between uracil and thymine is only one methyl group, the difference in the binding constant between them is more than 60 times. A mechanically controlled indicator displacement assay for aqueous glucose detection based on fluorescence resonance energy transfer was also reported [180].

**Figure 9:**
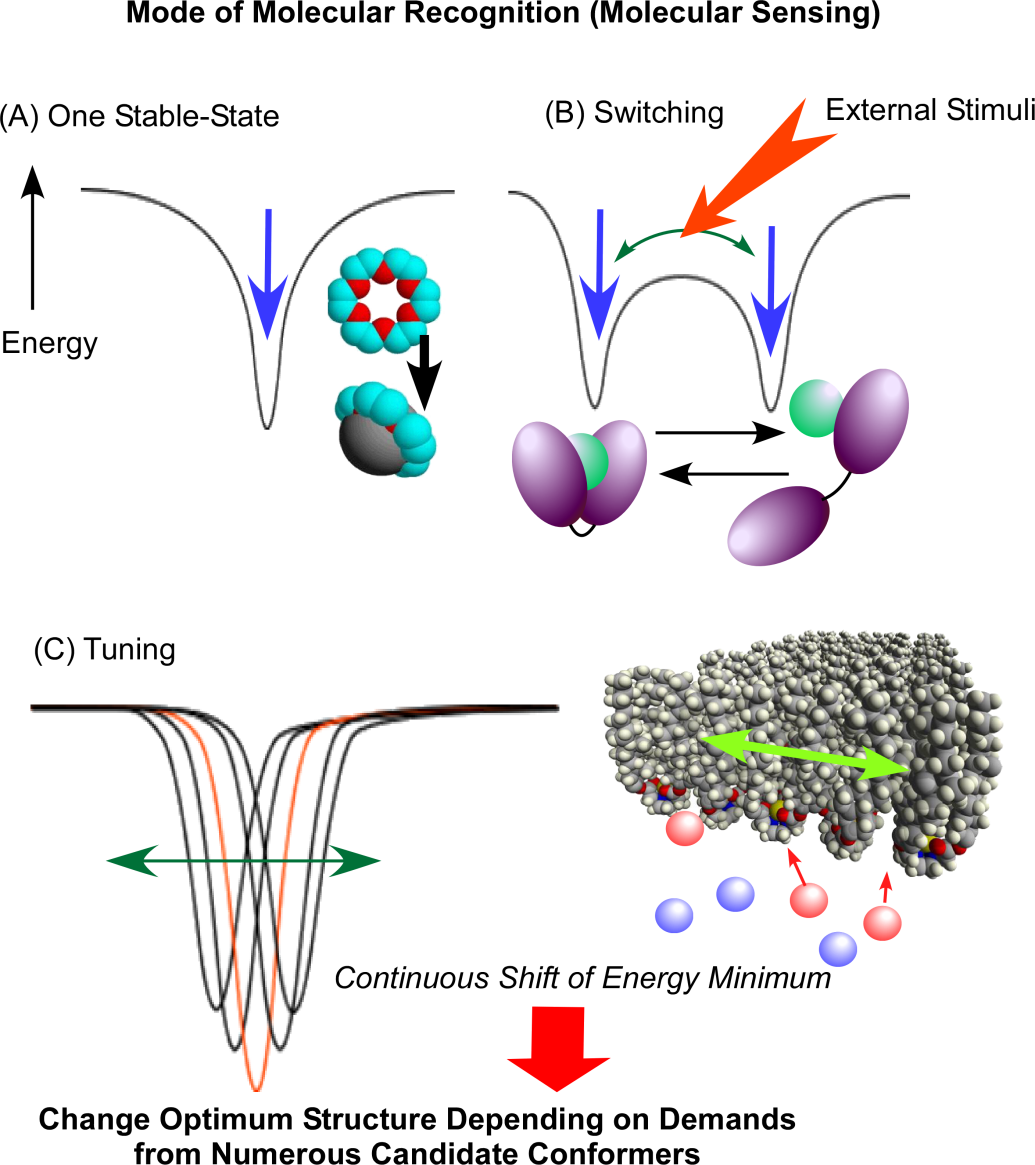
Mode of molecular recognition and sensing: (A) one most stable state between guest and host; (B) switching molecular binding by external stimuli; (c) tuning of receptor structures through continuously shifting molecular conformations for required demands. Adapted with permission from [52] copyright 2016, Springer Nature.

The mechanisms of molecular recognition and sensing are roughly summarized in Figure 9. The most basic mechanism (Figure 9) is considered to form the most stable state between the guest and host [181–183]. Shinkai and co-workers proposed a breakthrough approach to switch molecular recognition using photo-isomerization of an azobenzene moiety in a receptor structure (Figure 9) [184–185]. This mechanism creates two (or more) states with different binding energies that are controlled by external stimuli. This can also be regarded as the origination of molecular function control by external stimuli. It shares working principles with molecular machines which are usually operated by switching between several states [186–188]. Unlike these pioneering approaches, the mechanical tuning of receptor molecules at interfacial media considers numerous candidates from continuously shifting molecular conformations (Figure 9) [189–191]. This method may use all available possibilities of flexible molecular structures. This methodology has not been fully applied in practical sensing systems so far.

## Conclusion

This review article introduces several examples of recent advanced sensors, classified according to the sensing targets (chemical substance, physical condition and biological phenomena) in the first part. In the second part, the importance of nanoarchitectured motifs, such as nanoporous structures, ultrathin films, and unusual interfacial effects, for improved sensor performance is discussed. Most of the examples illustrate the crucial role of the nanostructure in sensor design. Although fine structural control used to be an important task in device miniaturization and integration in past approaches, the importance of precise nanoscale control for sensor materials is widely recognized in recent developments. Molecular sieving effects for better selectivity by well-designed nanoporous structures and effective carrier transport within a well-packed ultrathin monolayer of organic semiconductors have become clear in recent research examples. Of course, further efforts regarding nanoscale design of sensing materials for better performance and selectivity have to be made. In many cases, sensor advancements can be implemented with the nanoarchitectonics methodologies.

However, some important mechanisms such as huge enhancements of molecular recognition efficiency and molecular tuning capability at interfaces still remain as basic milestones and have not been applied in practical sensor applications to date. Further advancements of sensors can be made by exploitation of the various flexible and dynamic natures of sensing materials where various interactions and effects have to be harmonized similar to the nanoarchitectonics strategy. In addition, dynamic harmonization of the interactions is also commonly observed in biological processes and systems. As discussed in some reviews, interfacing between electronic devices and ionic biosystems [192] and biocompatible device design [193] are crucial for future sensor devices. Therefore, investigation on dynamic nanoarchitectonics for sensor devices could lead to further advancements in bio-friendly sensor devices.

Of course, all important sensor activities cannot be described in this review. For example, sensors based on various advanced physical mechanisms such as plasmonic [194], dielectric sensing [195], surface-enhanced Raman scattering [196], Fabry–Pérot-based intraocular pressure [197], and/or novel nanostructured materials with exotic properties [198] undoubtedly have important contributions. In addition, mass-sensitive sensors, quartz and crystal microbalance [199] are are useful for many substances because mass changes and alteration of viscoelasticity such as phase transition [200] are common over all materials. Once developed, these technologies have to be translated into real-world applications for potential impact on daily life. A roadmap for this technology transfer cannot be easily predicted, but should include important factors such as miniaturization, wearable features, scalability, reliability, and sampling of analytes. This roadmap would be shortened by using new types of materials such as two-dimensional materials [201–204] and through introducing new methodologies such as mass-data analyses and machine learning [205–206]. Another important factor to accelerate progress would be process integration of top down microfabrication and bottom up self-organization to bridge materials and systems over a wide scale range. To combine all of these techniques and functional materials, the concept of nanoarchitectonics becomes a crucial bridge in this roadmap.

## References

[1] Imran M, Motta N, Shafiei M (2018). Beilstein J Nanotechnol.

[2] Zhang Y, Yuan S, Day G, Wang X, Yang X, Zhou H-C (2018). Coord Chem Rev.

[3] Dey A (2018). Mater Sci Eng, B.

[4] Sun D, Luo Y, Debliquy M, Zhang C (2018). Beilstein J Nanotechnol.

[5] Datta K K R, Reddy B V S, Ariga K, Vinu A (2010). Angew Chem, Int Ed.

[6] Chaikittisilp W, Ariga K, Yamauchi Y (2013). J Mater Chem A.

[7] Jeevanandam J, Barhoum A, Chan Y S, Dufresne A, Danquah M K (2018). Beilstein J Nanotechnol.

[8] Irie M, Morimoto M (2018). Bull Chem Soc Jpn.

[9] Ishihara S, Labuta J, Nakanishi T, Tanaka T, Kataura H (2017). ACS Sens.

[10] Sarikhani Z, Manoochehri M (2017). Bull Chem Soc Jpn.

[11] Rasheed T, Bilal M, Nabeel F, Iqbal H M N, Li C, Zhou Y (2018). Sci Total Environ.

[12] Acharya R, Naik B, Parida K (2018). Beilstein J Nanotechnol.

[13] Zhang X, Jia S, Song J, Wu S, Han X (2018). Bull Chem Soc Jpn.

[14] Shak K P Y, Pang Y L, Mah S K (2018). Beilstein J Nanotechnol.

[15] Hou J, Inganäs O, Friend R H, Gao F (2018). Nat Mater.

[16] Miyasaka T (2018). Bull Chem Soc Jpn.

[17] Guo D, Shibuya R, Akiba C, Saji S, Kondo T, Nakamura J (2016). Science.

[18] Chaikittisilp W, Hu M, Wang H, Huang H-S, Fujita T, Wu K C-W, Chen L-C, Yamauchi Y, Ariga K (2012). Chem Commun.

[19] Watanabe M, Dokko K, Ueno K, Thomas M L (2018). Bull Chem Soc Jpn.

[20] Kawai T, Nakao S, Nishide H, Oyaizu K (2018). Bull Chem Soc Jpn.

[21] Yamamura A, Watanabe S, Uno M, Mitani M, Mitsui C, Tsurumi J, Isahaya N, Kanaoka Y, Okamoto T, Takeya J (2018). Sci Adv.

[22] Ulaganathan R K, Chang Y-H, Wang D-Y, Li S-S (2018). Bull Chem Soc Jpn.

[23] Watanabe Y, Sasabe H, Kido J (2019). Bull Chem Soc Jpn.

[24] Ariga K, Lvov Y M, Kawakami K, Ji Q, Hill J P (2011). Adv Drug Delivery Rev.

[25] Li B L, Setyawati M I, Chen L, Xie J, Ariga K, Lim C-T, Garaj S, Leong D T (2017). ACS Appl Mater Interfaces.

[26] He H, Xu B (2018). Bull Chem Soc Jpn.

[27] Kumar J, Liz-Marzán L M (2019). Bull Chem Soc Jpn.

[28] Povie G, Segawa Y, Nishihara T, Miyauchi Y, Itami K (2017). Science.

[29] Sun Z, Matsuno T, Isobe H (2018). Bull Chem Soc Jpn.

[30] Sun Z, Ikemoto K, Fukunaga T M, Koretsune T, Arita R, Sato S, Isobe H (2019). Science.

[31] Ruiz-Hitzky E, Darder M, Aranda P, Ariga K (2010). Adv Mater (Weinheim, Ger).

[32] Seiki N, Shoji Y, Kajitani T, Ishiwari F, Kosaka A, Hikima T, Takata M, Someya T, Fukushima T (2015). Science.

[33] Sawada T, Serizawa T (2018). Bull Chem Soc Jpn.

[34] Xing R, Yuan C, Li S, Song J, Li J, Yan X (2018). Angew Chem, Int Ed.

[35] Dhiman S, George S J (2018). Bull Chem Soc Jpn.

[36] Ringleb F, Andree S, Heidmann B, Bonse J, Eylers K, Ernst O, Boeck T, Schmid M, Krüger J (2018). Beilstein J Nanotechnol.

[37] Mori T, Tanaka H, Dalui A, Mitoma N, Suzuki K, Matsumoto M, Aggarwal N, Patnaik A, Acharya S, Shrestha L K (2018). Angew Chem, Int Ed.

[38] Chen R, Kang J, Kang M, Lee H, Lee H (2018). Bull Chem Soc Jpn.

[39] Sung B, Kim M-H (2018). Beilstein J Nanotechnol.

[40] Asanuma H, Murayama K, Kamiya Y, Kashida H (2018). Bull Chem Soc Jpn.

[41] Ariga K, Jia X, Song J, Hsieh C-T, Hsu S-h (2019). ChemNanoMat.

[42] Einaga Y (2018). Bull Chem Soc Jpn.

[43] Kitamori T (2019). Bull Chem Soc Jpn.

[44] Xie Y, Ding Y, Li X, Wang C, Hill J P, Ariga K, Zhang W, Zhu W (2012). Chem Commun.

[45] Izawa H, Wada M, Nishino S, Sumita M, Fujita T, Morihashi K, Ifuku S, Morimoto M, Saimoto H (2018). Bull Chem Soc Jpn.

[46] Wang Y, Michinobu T (2017). Bull Chem Soc Jpn.

[47] Maduraiveeran G, Sasidharan M, Ganesan V (2018). Biosens Bioelectron.

[48] Huang R, He N, Li Z (2018). Biosens Bioelectron.

[49] Ferhan A R, Jackman J A, Park J H, Cho N-J, Kim D-H (2018). Adv Drug Delivery Rev.

[50] Li B L, Wang J, Gao Z F, Shi H, Zou H L, Ariga K, Leong D T (2019). Mater Horiz.

[51] Ariga K, Ji Q, Nakanishi W, Hill J P, Aono M (2015). Mater Horiz.

[52] Ariga K, Minami K, Ebara M, Nakanishi J (2016). Polym J.

[53] Ishihara S, Labuta J, Van Rossom W, Ishikawa D, Minami K, Hill J P, Ariga K (2014). Phys Chem Chem Phys.

[54] Ariga K, Ji Q, Hill J P, Bando Y, Aono M (2012). NPG Asia Mater.

[55] Ariga K, Nishikawa M, Mori T, Takeya J, Shrestha L K, Hill J P (2019). Sci Technol Adv Mater.

[56] Ariga K, Li M, Richards G J, Hill J P (2011). J Nanosci Nanotechnol.

[57] Ariga K, Li J, Fei J, Ji Q, Hill J P (2016). Adv Mater (Weinheim, Ger).

[58] Ramanathan M, Shrestha L K, Mori T, Ji Q, Hill J P, Ariga K (2013). Phys Chem Chem Phys.

[59] Nakanishi W, Minami K, Shrestha L K, Ji Q, Hill J P, Ariga K (2014). Nano Today.

[60] Ariga K, Malgras V, Ji Q, Zakaria M B, Yamauchi Y (2016). Coord Chem Rev.

[61] Sakakibara K, Hill J P, Ariga K (2011). Small.

[62] Ariga K, Vinu A, Yamauchi Y, Ji Q, Hill J P (2012). Bull Chem Soc Jpn.

[63] Ariga K, Yamauchi Y, Rydzek G, Ji Q, Yonamine Y, Wu K C-W, Hill J P (2014). Chem Lett.

[64] Ariga K, Watanabe S, Mori T, Takeya J (2018). NPG Asia Mater.

[65] Sang Y, Liu M (2019). Mol Syst Des Eng.

[66] Abe H, Liu J, Ariga K (2016). Mater Today.

[67] Ariga K, Ishihara S, Abe H (2016). CrystEngComm.

[68] Kim J, Kim J H, Ariga K (2017). Joule.

[69] Khan A H, Ghosh S, Pradhan B, Dalui A, Shrestha L K, Acharya S, Ariga K (2017). Bull Chem Soc Jpn.

[70] Ariga K, Yamauchi Y, Ji Q, Yonamine Y, Hill J P (2014). APL Mater.

[71] Pandeeswar M, Senanayak S P, Govindaraju T (2016). ACS Appl Mater Interfaces.

[72] Ariga K, Ji Q, McShane M J, Lvov Y M, Vinu A, Hill J P (2012). Chem Mater.

[73] Ariga K, Ji Q, Mori T, Naito M, Yamauchi Y, Abe H, Hill J P (2013). Chem Soc Rev.

[74] Komiyama M, Yoshimoto K, Sisido M, Ariga K (2017). Bull Chem Soc Jpn.

[75] Ariga K, Leong D T, Mori T (2018). Adv Funct Mater.

[76] Ariga K, Kawakami K, Ebara M, Kotsuchibashi Y, Ji Q, Hill J P (2014). New J Chem.

[77] Pandey A P, Girase N M, Patil M D, Patil P O, Patil D A, Deshmukh P K (2014). J Nanosci Nanotechnol.

[78] Zhao L, Zou Q, Yan X (2019). Bull Chem Soc Jpn.

[79] Ariga K, Minami K, Shrestha L K (2016). Analyst.

[80] Jackman J A, Cho N-J, Nishikawa M, Yoshikawa G, Mori T, Shrestha L K, Ariga K (2018). Chem – Asian J.

[81] Aono M, Ariga K (2016). Adv Mater (Weinheim, Ger).

[82] Ariga K (2017). Mater Chem Front.

[83] Osica I, Imamura G, Shiba K, Ji Q, Shrestha L K, Hill J P, Kurzydłowski K J, Yoshikawa G, Ariga K (2017). ACS Appl Mater Interfaces.

[84] Osica I, Melo A F A A, Imamura G, Shiba K, Ji Q, Hill J P, Crespilho F N, Kurzydłowski K J, Yoshikawa G, Ariga K (2017). J Nanosci Nanotechnol.

[85] Tang K, Song Z, Tang Q, Tian H, Tong Y, Liu Y (2018). IEEE Electron Device Lett.

[86] Liu Y, Wang Y, Ikram M, Lv H, Chang J, Li Z, Ma L, Rehman A U, Lu G, Chen J (2018). ACS Sens.

[87] Rodlamul P, Tamura S, Imanaka N (2019). Bull Chem Soc Jpn.

[88] Sasaki J, Suzuki M, Hanabusa K (2018). Bull Chem Soc Jpn.

[89] Kondo S-i, Sato K, Matsuta Y, Osawa K (2018). Bull Chem Soc Jpn.

[90] Mulla M Y, Tuccori E, Magliulo M, Lattanzi G, Palazzo G, Persaud K, Torsi L (2015). Nat Commun.

[91] Park K M, Kim J, Ko Y H, Ahn Y, Murray J, Li M, Shrinidhi A, Kim K (2018). Bull Chem Soc Jpn.

[92] Akamatsu M, Komatsu H, Matsuda A, Mori T, Nakanishi W, Sakai H, Hill J P, Ariga K (2017). Bull Chem Soc Jpn.

[93] Ruiz-Hitzky E, Gómez-Avilés A, Darder M, Aranda P (2018). Bull Chem Soc Jpn.

[94] Li P, Zhang D, Jiang C, Zong X, Cao Y (2017). Biosens Bioelectron.

[95] Spanu A, Viola F, Lai S, Cosseddu P, Ricci P C, Bonfiglio A (2017). Org Electron.

[96] Lee S, Reuveny A, Reeder J, Lee S, Jin H, Liu Q, Yokota T, Sekitani T, Isoyama T, Abe Y (2016). Nat Nanotechnol.

[97] Cao R, Pu X, Du X, Yang W, Wang J, Guo H, Zhao S, Yuan Z, Zhang C, Li C (2018). ACS Nano.

[98] Askari H, Hashemi E, Khajepour A, Khamesee M B, Wang Z L (2018). Nano Energy.

[99] Ren Z, Ding Y, Nie J, Wang F, Xu L, Lin S, Chen X, Wang Z L (2019). ACS Appl Mater Interfaces.

[100] Wang J, Ding W, Pan L, Wu C, Yu H, Yang L, Liao R, Wang Z L (2018). ACS Nano.

[101] Melzer M, Kaltenbrunner M, Makarov D, Karnaushenko D, Karnaushenko D, Sekitani T, Someya T, Schmidt O G (2015). Nat Commun.

[102] Piro B, Wang D, Benaoudia D, Tibaldi A, Anquetin G, Noël V, Reisberg S, Mattana G, Jackson B (2017). Biosens Bioelectron.

[103] Komiyama M, Mori T, Ariga K (2018). Bull Chem Soc Jpn.

[104] Lai Y, Deng Y, Yang G, Li S, Zhang C, Liu X (2018). J Biomed Nanotechnol.

[105] Takeuchi T, Sunayama H (2018). Chem Commun.

[106] Zhang L, Wang G, Xiong C, Zheng L, He J, Ding Y, Lu H, Zhang G, Cho K, Qiu L (2018). Biosens Bioelectron.

[107] Sekitani T, Yokota T, Kuribara K, Kaltenbrunner M, Fukushima T, Inoue Y, Sekino M, Isoyama T, Abe Y, Onodera H (2016). Nat Commun.

[108] Hempel F, Law J K-Y, Nguyen T C, Munief W, Lu X, Pachauri V, Susloparova A, Vu X T, Ingebrandt S (2017). Biosens Bioelectron.

[109] Yeung S Y, Gu X, Tsang C M, Tsao S W, Hsing I-m (2019). Sens Actuators, A.

[110] Pulikkathodi A K, Sarangadharan I, Chen Y-H, Lee G-Y, Chyi J-I, Lee G-B, Wang Y-L (2018). Lab Chip.

[111] Braendlein M, Pappa A-M, Ferro M, Lopresti A, Acquaviva C, Mamessier E, Malliaras G G, Owens R M (2017). Adv Mater (Weinheim, Ger).

[112] Cherumukkil S, Vedhanarayanan B, Das G, Praveen V K, Ajayaghosh A (2018). Bull Chem Soc Jpn.

[113] Shimizu T (2018). Bull Chem Soc Jpn.

[114] Liu X, Riess J G, Krafft M P (2018). Bull Chem Soc Jpn.

[115] Hu M, Reboul J, Furukawa S, Torad N L, Ji Q, Srinivasu P, Ariga K, Kitagawa S, Yamauchi Y (2012). J Am Chem Soc.

[116] Chaikittisilp W, Torad N L, Li C, Imura M, Suzuki N, Ishihara S, Ariga K, Yamauchi Y (2014). Chem – Eur J.

[117] Malgras V, Ji Q, Kamachi Y, Mori T, Shieh F-K, Wu K C-W, Ariga K, Yamauchi Y (2015). Bull Chem Soc Jpn.

[118] Saptiama I, Kaneti Y V, Oveisi H, Suzuki Y, Tsuchiya K, Takai K, Sakae T, Pradhan S, Hossain M S A, Fukumitsu N (2018). Bull Chem Soc Jpn.

[119] Sai-Anand G, Sivanesan A, Benzigar M R, Singh G, Gopalan A-I, Baskar A V, Ilbeygi H, Ramadass K, Kambala V, Vinu A (2019). Bull Chem Soc Jpn.

[120] Shrestha L K, Shrestha R G, Yamauchi Y, Hill J P, Nishimura T, Miyazawa K, Kawai T, Okada S, Wakabayashi K, Ariga K (2015). Angew Chem, Int Ed.

[121] Bairi P, Minami K, Nakanishi W, Hill J P, Ariga K, Shrestha L K (2016). ACS Nano.

[122] Bairi P, Minami K, Hill J P, Ariga K, Shrestha L K (2017). ACS Nano.

[123] Ariga K, Vinu A, Ji Q, Ohmori O, Hill J P, Acharya S, Koike J, Shiratori S (2008). Angew Chem, Int Ed.

[124] Ariga K, Vinu A, Miyahara M, Hill J P, Mori T (2007). J Am Chem Soc.

[125] Kosaki Y, Izawa H, Ishihara S, Kawakami K, Sumita M, Tateyama Y, Ji Q, Krishnan V, Hishita S, Yamauchi Y (2013). ACS Appl Mater Interfaces.

[126] Ji Q, Yoon S B, Hill J P, Vinu A, Yu J-S, Ariga K (2009). J Am Chem Soc.

[127] Torad N L, Hu M, Ishihara S, Sukegawa H, Belik A A, Imura M, Ariga K, Sakka Y, Yamauchi Y (2014). Small.

[128] Mei L, Shi W-q, Chai Z-f (2018). Bull Chem Soc Jpn.

[129] Li J, Wang X, Zhao G, Chen C, Chai Z, Alsaedi A, Hayat T, Wang X (2018). Chem Soc Rev.

[130] Azhar A, Li Y, Cai Z, Zakaria M B, Masud M K, Hossain M S A, Kim J, Zhang W, Na J, Yamauchi Y (2019). Bull Chem Soc Jpn.

[131] Chen L, Ye J-W, Wang H-P, Pan M, Yin S-Y, Wei Z-W, Zhang L-Y, Wu K, Fan Y-N, Su C-Y (2017). Nat Commun.

[132] Tian H, Fan H, Li M, Ma L (2016). ACS Sens.

[133] Takimiya K, Nakano M (2018). Bull Chem Soc Jpn.

[134] Suda M (2018). Bull Chem Soc Jpn.

[135] Ariga K, Yamauchi Y, Mori T, Hill J P (2013). Adv Mater (Weinheim, Ger).

[136] Seki T (2018). Bull Chem Soc Jpn.

[137] Ariga K, Mori T, Li J (2019). Langmuir.

[138] Ji Q, Honma I, Paek S-M, Akada M, Hill J P, Vinu A, Ariga K (2010). Angew Chem, Int Ed.

[139] Rydzek G, Ji Q, Li M, Schaaf P, Hill J P, Boulmedais F, Ariga K (2015). Nano Today.

[140] Ji Q, Qiao X, Liu X, Jia H, Yu J-S, Ariga K (2018). Bull Chem Soc Jpn.

[141] Rodrigues V C, Moraes M L, Soares J C, Soares A C, Sanfelice R, Deffune E, Oliveira O N (2018). Bull Chem Soc Jpn.

[142] Furusawa H, Ichimura Y, Harada S, Uematsu M, Xue S, Nagamine K, Tokito S (2018). Bull Chem Soc Jpn.

[143] Endo S, Kato R, Sawada K, Hattori T (2018). Bull Chem Soc Jpn.

[144] Chen H, Dong S, Bai M, Cheng N, Wang H, Li M, Du H, Hu S, Yang Y, Yang T (2015). Adv Mater (Weinheim, Ger).

[145] Peng B, Huang S, Zhou Z, Chan P K L (2017). Adv Funct Mater.

[146] Ariga K, Kunitake T (1998). Acc Chem Res.

[147] Ariga K, Ito H, Hill J P, Tsukube H (2012). Chem Soc Rev.

[148] Onda M, Yoshihara K, Koyano H, Ariga K, Kunitake T (1996). J Am Chem Soc.

[149] Springs B, Haake P (1977). Bioorg Chem.

[150] Sasaki D Y, Kurihara K, Kunitake T (1991). J Am Chem Soc.

[151] Sasaki D Y, Kurihara K, Kunitake T (1992). J Am Chem Soc.

[152] Sakurai M, Tamagawa H, Furuki T, Inoue Y, Ariga K, Kunitake T (1995). Chem Lett.

[153] Sakurai M, Tamagawa H, Inoue Y, Ariga K, Kunitake T (1997). J Phys Chem B.

[154] Tamagawa H, Sakurai M, Inoue Y, Ariga K, Kunitake T (1997). J Phys Chem B.

[155] Ariga K (2016). ChemNanoMat.

[156] Ikeura Y, Kurihara K, Kunitake T (1991). J Am Chem Soc.

[157] Cha X, Ariga K, Onda M, Kunitake T (1995). J Am Chem Soc.

[158] Cha X, Ariga K, Kunitake T (1996). J Am Chem Soc.

[159] Ariga K, Kamino A, Cha X, Kunitake T (1999). Langmuir.

[160] Kurihara K, Ohto K, Tanaka Y, Aoyama Y, Kunitake T (1991). J Am Chem Soc.

[161] Ariga K, Isoyama K, Hayashida O, Aoyama Y, Okahata Y (1998). Chem Lett.

[162] Kurihara K, Ohto K, Honda Y, Kunitake T (1991). J Am Chem Soc.

[163] Kawahara T, Kurihara K, Kunitake T (1992). Chem Lett.

[164] Taguchi K, Ariga K, Kunitake T (1995). Chem Lett.

[165] Ariga K, Kamino A, Koyano H, Kunitake T (1997). J Mater Chem.

[166] Oishi Y, Torii Y, Kato T, Kuramori M, Suehiro K, Ariga K, Taguchi K, Kamino A, Koyano H, Kunitake T (1997). Langmuir.

[167] Ariga K, Mori T, Hill J P (2012). Adv Mater (Weinheim, Ger).

[168] Ariga K, Mori T, Ishihara S, Kawakami K, Hill J P (2014). Chem Mater.

[169] Ariga K, Mori T, Nakanishi W, Hill J P (2017). Phys Chem Chem Phys.

[170] Ishikawa D, Mori T, Yonamine Y, Nakanishi W, Cheung D L, Hill J P, Ariga K (2015). Angew Chem, Int Ed.

[171] Mori T, Ishikawa D, Yonamine Y, Fujii Y, Hill J P, Ichinose I, Ariga K, Nakanishi W (2017). ChemPhysChem.

[172] Ariga K, Terasaka Y, Sakai D, Tsuji H, Kikuchi J-i (2000). J Am Chem Soc.

[173] Ariga K, Nakanishi T, Terasaka Y, Tsuji H, Sakai D, Kikuchi J-i (2005). Langmuir.

[174] Mori T, Komatsu H, Sakamoto N, Suzuki K, Hill J P, Matsumoto M, Sakai H, Ariga K, Nakanishi W (2018). Phys Chem Chem Phys.

[175] Mori T, Chin H, Kawashima K, Ngo H T, Cho N-J, Nakanishi W, Hill J P, Ariga K (2019). ACS Nano.

[176] Michinobu T, Shinoda S, Nakanishi T, Hill J P, Fujii K, Player T N, Tsukube H, Ariga K (2006). J Am Chem Soc.

[177] Michinobu T, Shinoda S, Nakanishi T, Hill J P, Fujii K, Player T N, Tsukube H, Ariga K (2011). Phys Chem Chem Phys.

[178] Mori T, Okamoto K, Endo H, Hill J P, Shinoda S, Matsukura M, Tsukube H, Suzuki Y, Kanekiyo Y, Ariga K (2010). J Am Chem Soc.

[179] Mori T, Okamoto K, Endo H, Sakakibara K, Hill J P, Shinoda S, Matsukura M, Tsukube H, Suzuki Y, Kanekiyo Y (2011). Nanoscale Res Lett.

[180] Sakakibara K, Joyce L A, Mori T, Fujisawa T, Shabbir S H, Hill J P, Anslyn E V, Ariga K (2012). Angew Chem, Int Ed.

[181] Lehn J-M (1988). Angew Chem, Int Ed Engl.

[182] Pedersen C J (1988). Angew Chem, Int Ed Engl.

[183] Cram D J (1988). Angew Chem, Int Ed Engl.

[184] Shinkai S, Manabe O (1984). Top Curr Chem.

[185] Shinkai S, Ikeda M, Sugasaki A, Takeuchi M (2001). Acc Chem Res.

[186] Feringa B L (2017). Angew Chem, Int Ed.

[187] Sauvage J-P (2017). Angew Chem, Int Ed.

[188] Stoddart J F (2017). Angew Chem, Int Ed.

[189] Ariga K (2016). Anal Sci.

[190] Shirai Y, Minami K, Nakanishi W, Yonamine Y, Joachim C, Ariga K (2016). Jpn J Appl Phys.

[191] Shrestha L K, Mori T, Ariga K (2018). Curr Opin Colloid Interface Sci.

[192] Nishizawa M (2018). Bull Chem Soc Jpn.

[193] Stauss S, Honma I (2018). Bull Chem Soc Jpn.

[194] Anker J N, Hall W P, Lyandres O, Shah N C, Zhao J, Van Duyne R P (2008). Nat Mater.

[195] Tittl A, Leitis A, Liu M, Yesilkoy F, Choi D-Y, Neshev D N, Kivshar Y S, Altug H (2018). Science.

[196] Vo-Dinh T (1995). Sens Actuators, B.

[197] Narasimhan V, Siddique R H, Lee J O, Kumar S, Ndjamen B, Du J, Hong N, Sretavan D, Choo H (2018). Nat Nanotechnol.

[198] Sreekanth K V, Alapan Y, ElKabbash M, Ilker E, Hinczewski M, Gurkan U A, De Luca A, Strangi G (2016). Nat Mater.

[199] Emir Diltemiz S, Keçili R, Ersöz A, Say R (2017). Sensors.

[200] Okahata Y, Kimura K, Ariga K (1989). J Am Chem Soc.

[201] Tan S M, Poh H L, Sofer Z, Pumera M (2013). Analyst.

[202] Marvan P, Mazánek V, Sofer Z (2019). Nanoscale.

[203] Kang J, Wells S A, Sangwan V K, Lam D, Liu X, Luxa J, Sofer Z, Hersam M C (2018). Adv Mater (Weinheim, Ger).

[204] Lee C M, Jin C H, Ahn C H, Cho H K, Lim J H, Hwang S M, Joo J (2019). Bull Chem Soc Jpn.

[205] Liakos K, Busato P, Moshou D, Pearson S, Bochtis D (2018). Sensors.

[206] Fonollosa J, Solórzano A, Marco S (2018). Sensors.

